# Formulation and Preparation of Losartan-Potassium-Loaded Controlled-Release Matrices Using Ethocel Grade 10 to Establish a Correlation between In Vitro and In Vivo Results

**DOI:** 10.3390/pharmaceutics16020186

**Published:** 2024-01-28

**Authors:** Kamran Ahmad Khan, Ashfaq Ahmad, Carlotta Marini, Mario Nicotra, Alessandro Di Cerbo, Fazal-Ur-Rehman, Naveed Ullah, Gul Majid Khan

**Affiliations:** 1 Gomal Centre of Pharmaceutical Sciences, Faculty of Pharmacy, Gomal University, Dera Ismail Khan P.O. Box 29050, Pakistan; fazal@gu.edu.pk; 2 Riphah Institute of Pharmaceutical Sciences, Riphah International University, Gulberg Greens Campus, Islamabad P.O. Box 44000, Pakistan; ashfaq.ahmed@riphah.edu.pk; 3 School of Biosciences and Veterinary Medicine, University of Camerino, 62024 Matelica, Italy; carlotta.marini@unicam.it (C.M.); mario.nicotra@studenti.unicam.it (M.N.); 4 Department of Pharmacy, University of Swabi, Swabi P.O. Box 23430, Pakistan; dr.naveedullah@uoswabi.edu.pk; 5 Islamia College University, Peshawar P.O. Box 25120, Pakistan; gulmajeedkhan@yahoo.com

**Keywords:** controlled release, losartan potassium, Ethocel grade 10, in vitro-in vivo correlation

## Abstract

In the current study, matrices of losartan potassium were formulated with two different polymers (Ethocel 10 premium and Ethocel 10FP premium), along with a filler and a lubricant, at different drug-to-polymer *w*/*w* ratios (10:3, 10:4, and 10:5). The matrices were tested by the direct compression method, and their hardness, diameter, thickness, friability, weight variation, content uniformity, and in vitro dissolution tests were assessed to determine 24-h drug release rates. The matrices with Ethocel 10 FP at a 10:4 ratio exhibited pseudo-zero-order kinetics (n-value of 0.986), while the dissolution data of the test matrices and reference tablets did not match. The new test-optimized matrices were also tested in rabbits, and their pharmacokinetic parameters were investigated: half-life (11.78 ± 0.018 h), Tmax (2.105 ± 1.131 h), C_max_ (205.98 ± 0.321 μg/mL), AUC_o_ (5931.10 ± 1.232 μg·h/mL), AUC_o-inf_ (7348.46 ± 0.234 μg·h/mL), MRT_o-48h_ (17.34 ± 0.184 h), and Cl (0.002 ± 0.134 mL/min). A correlation value of 0.985 between the in vitro and in vivo results observed for the test-optimized matrices was observed, indicating a level-A correlation between the percentage of the drug released in vitro and the percentage of the drug absorbed in vivo. The matrices might improve patient compliance with once-a-day dosing and therapeutic outcomes.

## 1. Introduction

Drug delivery via the oral route is the most popular method. Tablets, the most widely used oral format on the market, are chosen by patients and medical professionals. Conventional formulations must be given in various doses during long-term therapy for the management of chronic illnesses, which has several drawbacks. Developing dosage forms that control the drug’s entry into the body at the target site and function for a long period has been shown to be a suitable approach to optimize therapeutic outcomes [1]. Such systems enable the release of drugs to achieve the desired therapeutic response. Traditional drug delivery systems have fluctuating drug levels in plasma and might affect the therapeutic level of the drug. The therapeutic process can only be rendered useful with an efficient delivery mechanism.

Furthermore, to achieve maximum efficacy and safety, the drug must be delivered to the target site as precisely as possible at a specific and controlled rate. Controlled drug delivery systems are being developed to address the problems associated with conventional drug delivery. Over the past two decades, controlled drug delivery systems have progressed tremendously from macro- and nanoscale systems to intelligent, targeted delivery [2]. Controlled drug delivery systems can maintain drug levels within a particular range, necessitate fewer doses, ensure the medicine is used to its full potential, and promote patient compliance. A high blood level of the drug over an extended period was the aim of many of the earliest controlled-release systems’ delivery profiles. For the oral route, matrices have been chosen as the most likely mode of prolongation of drug release. The typical components of an oral controlled drug delivery system are either a drug core coated with a film (also indicated as a membrane) or a matrix in which the drug is dispersed [3].

A matrix is one of the approaches to controlled-release systems, which is an oral solid dosage form that maintains stable plasma drug concentrations and release rates to produce long-term therapeutic activity. Such controlled-release systems are designed for short-lived drugs with frequent dosing [4]. A matrix is an oral solid dosage form in which the drug or active ingredient is uniformly dissolved or dispersed throughout the hydrophilic or hydrophobic polymeric matrix, which serves as a release rate retardant. In matrices, matrix-forming materials are often swellable hydrophilic or non-swellable hydrophobic polymers, and the polymer properties affect the drug release rate from the matrix through diffusion, permeation, and dissolution. For the drug to be released, it must first dissolve. Therefore, the rate at which water permeates the matrix is directly related to the drug’s ability to dissolve, diffuse out of the matrix and diffuse throughout the solution. Therefore, the matrix’s diffusion path length influences the drug’s release rate and subsequent absorption. Diffusion occurs when the drug moves from an area of high concentration within the matrix core to an area of low concentration in the surrounding medium [5].

Excipients are inert constituents incorporated in dosage forms other than drugs and added to dosage forms for different uses, such as improving stability, enhancing bioavailability, helping in the preparation procedure, improving the appearance or taste, and easing the delivery or administration of the active ingredient [6]. Excipients of an oral drug delivery system are classified based on their functions, including binders (binding agent), diluents (bulking agent), lubricants (reduce friction during ejection at the point where a tablet’s surface meets the die wall to lessen wear on punches and dies), disintegrating agents, plasticizers, and polymers [7]. Polymers are used in oral controlled-release systems as matrix-forming materials to control drug release rates. Polymers have various pharmaceutical applications, including as binders, enhancing viscosity, and improving flow for liquids, suspensions, and emulsions. Polymers can be employed as film coatings to improve drug stability, change drug release properties, and mask a drug’s disagreeable taste. Polymer types include natural polymers (e.g., dextrin, arginine, chitosan, polysaccharides), synthetic polymers (e.g., poly(N-isopropyl acrylamide)s, poly (2-hydroxyethyl methacrylate), poly(ethylenimine)s), and biodegradable, bioabsorbable, and dendritic polymers [8]. Ethocel ethyl cellulose polymers are colorless, odorless, tasteless, and non-caloric. Ethocel dissolves in various solvents, such as natural oils, chlorinated solvents, and aliphatic alcohols. It is practically insoluble in water, propylene glycol, and glycerin. These multifunctional, water-insoluble, and organosoluble polymers are used in various pharmaceutical and specialty uses. They function as binders, tougheners, maskers, time-release agents, flexible film formers, rheology modifiers, and water barriers. In Ethocel grade 10, there are Ethocel 10 premium (average particle size 375 µm) and Ethocel 10 FP premium (average particle size 6.4 µm) [9]. Previously, different studies were reported using different polymers to prepare their drug matrices. Sustained-release matrices of losartan potassium were prepared using the polymers Eudragit RLPO, Eudragit RSPO, and ethyl cellulose, and they found extended release of the drug ethyl cellulose when used in combination with polymers used alone [10]. Sustained-release matrices of losartan potassium were prepared with xanthan gum, HPMC, and ethyl cellulose and evaluated for in vitro drug release [11]. Ethocel grade 100 alone was used to prepare the losartan potassium matrices [12], and Ethocel 7 combined with Carbopol 934 was used as a rate-retarding polymer [13]. The polymer Ethocel grade 10 combined with Carbopol was used to prepare controlled-release matrices [14]. Sustained-release matrices of losartan potassium were formulated using hydroxymethyl propyl cellulose (HPMC) K100M and xanthan gum in combination with a direct compression technique [15]. Controlled-release matrices of losartan potassium were prepared with different polymers: acacia gum, copal gum, hydroxypropyl methylcellulose K100 (HPMC K100), carboxy methyl ethyl cellulose (CMEC), and Eudragit RL 100, alone and in combination [16]. The current study used Ethocel grade 10 (Ethocel 10 premium and Ethocel 10 FP premium) polymers, which were used alone to produce controlled-release losartan potassium matrices and to check the effects of their particle size on drug release rates.

The co-processed excipients or co-excipients are blends of two or more compendial or non-compendial excipients developed to physically modify their properties in a manner that is not attainable by simple physical mixing and without a significant chemical change. They help to overcome deficits arising with the use of single general-grade excipients. The co-excipients have higher functionalities than the individual excipients, like better flow properties, reduced lubricant sensitivity, and compressibility [17]. Hydroxypropyl methylcellulose (HPMC) is a white or milky white, tasteless, odorless, fibrous powder or granule with a weight loss on drying that does not exceed 10%, soluble in cold but not hot water, swelling slowly in hot water, and forming a viscous colloidal solution, which becomes a solution when cooled and becomes a gel when heated. It is soluble in a mixed solvent of methanol and methyl chloride [18]. Carboxymethylcellulose is a white, creamy powder that is tasteless and odorless. It is a linear, non-toxic, anionic, long-chain, and inexpensive water-soluble anionic polysaccharide with good film-forming ability. Starch is commonly used in different oral controlled-release systems in the pharmaceutical industry since it is a non-toxic, cheap, and biocompatible co-excipient [19]. In the current study, along with the drug and excipients, co-excipients such as carboxymethylcellulose (CMC), hydroxypropyl methylcellulose (HPMC), and starch were used (10% of the filler) to check their effects on drug release rates.

Losartan potassium, a non-peptide molecule, is chemically termed 2-butyl-4-chloro-1-[p-(o-1H-tetrazol-5-ylphenyl)benzyl]imidazole-5-methanol monopotassium salt. Its empirical formula is C_22_H_22_ClKN_6_O. It is a white–off-white crystalline powder whose melting point ranges from 263 to 265 °C. It is also freely soluble in water. The molecular mass of losartan potassium is 462.01. It belongs to Class III of the Biopharmaceutical Classification System (BCS) [20]. Losartan potassium is an oral angiotensin-II antagonist used to treat high blood pressure, primarily by blocking the AT1 receptor. It is a highly favored antihypertensive drug and prescription drug. Although the drug is highly soluble in water, its oral bioavailability is just 33%. This results from its inadequate absorption from the lower gastrointestinal tract and a 1.5–2.5 h plasma elimination half-life. Losartan potassium has short half-lives, so it would be preferable to administer it in controlled-release matrices and less frequently to keep the drug’s plasma levels stable for at least 18 to 26 h [21]. In the current study, losartan potassium, due to its short half-life (1.5–2.5 h) and regular dosing frequency, was added to controlled-release matrices using Ethocel grade 10 polymers to extend its half-life to 24 h, both in vitro and in vivo.

In previous studies, losartan potassium controlled-release systems, including matrices, have been evaluated in vitro and in vivo in attempts to extend the drug release rates. Sustained-release matrix pellets containing solid dispersions of losartan potassium were prepared using a hydrophilic polymer, PEG 6000, and mixed with Avicel^®^ PH 101. The optimized sustained-release pellets had a Tmax of 9.72 ± 2.22 h, compared to 2.11 ± 0.49 h in the case of the Cozaar^®^ immediate-release tablets, indicating a slower release of the drug from the pellets [22]. Bhanja et al. prepared and assessed mouth-dissolving/disintegrating tablets (MDTs) of losartan potassium to improve the dissolution rate and enhance the bioavailability. They found that the water absorption ratio, the wetting time, and the in vitro and in vivo disintegration times of B8 were 86.1%, 8 s, 18 s, and 25 s, respectively. The B8 released up to 99.21% in 2 min and was considered the best formulation [23].

Losartan potassium floating matrices were prepared using the direct compression method, with locust bean gum and HPMC K15M as polymers and sodium bicarbonate as a floating agent. The influence of the nature of the polymers was checked by preparing different formulations of matrices, and the drug amount (100 mg) was kept constant in the matrices. They found that matrices LPFT1 to LPFT9 sustained the drug release profiles, and the matrix LPFT4 sustained the drug release profile for 24 h, noting super case II transport diffusion drug release kinetics [24]. It was reported that the floating matrices of losartan potassium were formulated using different amounts of hydroxypropyl methylcellulose (HPMC-K4M) and karaya gum. The matrices were evaluated for various pre-compression and post-compression tests, in vitro drug release, and in vivo X-ray imaging in rabbits. Gastric X-ray imaging of formulation F9 (drug/HPMC-K4M/karaya gum/microcrystalline cellulose/sodium bicarbonate/magnesium stearate/lactose) revealed that the matrix was constantly floating in the rabbit’s stomach; therefore, it could extend the gastric retention time to more than 12 h [25]. It was reported in a study that sustained-release matrices of losartan potassium using natural polymers were developed by direct compression. The addition of natural polymers (xanthan and guar gum) to the matrix affected the release rate of the drug. In vitro release showed that the release rate decreased with an increased polymer ratio. The matrix SL1 released 76.08% of the drug in 12 h. The release mechanisms were determined using various kinetics models, and they noted that the drug was released by zero-order kinetics and followed super case II transport [26]. In different amounts, losartan potassium sustained-release matrices were developed by wet granulation using the hydrophilic polymer HPMC and natural polymers such as xanthan gum and guar gum. The hardness, weight uniformity, friability assay, and in vitro drug release were tested for the physiochemical uniformity of the matrices using both official and unofficial USP tests. The matrices showed optimal hardness, consistent weight uniformity, friability, assay, and extended drug rates for 10 h, and their drug release was no less than 70%. They concluded that the in vitro dissolution indicated that the matrices (F2, F3, F4, F5, and F8) had drug release profiles close to the theoretical release profile [27].

This work aimed to develop 200 mg controlled-release matrices of losartan potassium (100 mg) using the polymer Ethocel grade 10 (30 to 50 mg at drug-to-polymer ratios of 10:3, 10:4, and 10:5) as a rate-controlling agent, filler (44.1 to 69 mg), and lubricant (0.5%). These matrices were evaluated for physicochemical characteristics (thickness, diameter, friability, hardness, weight variation, assay, and in vitro dissolution) to achieve 24 h drug release rates, determine pharmacokinetic parameters (half-life, T_max_, C_max_, AUC_o_, AUC_o-inf_, MRT_0–48h,_ and Cl), and establish the optimized matrices’ correlation in vitro and in vivo.

## 2. Materials and Methods

### 2.1. Materials

Well & Well Pharma UK Ltd. (Islamabad, Pakistan) kindly gifted losartan potassium, Ethocel 10 premium (375 µm), and Ethocel 10 FP premium with a particle size of 6.4 µm (Dow Chemical Co., Midland, MI, USA). Magnesium stearate and spray-dried lactose were purchased from BDH Chemical Ltd. (Poole, UK); the co-excipients HPMC (white powder, 88 µm), CMC (88 µm), starch (88 µm), monobasic potassium phosphate, and sodium hydroxide (NaOH) were purchased from Sigma-Aldrich (St. Louis, MO, USA). Cardaktin^®^ tablets (Hygeia Pharmaceutical, Islamabad, Pakistan) were purchased from a local pharmaceutical market as immediate-release tablets containing 100 mg of losartan potassium. Losartan potassium controlled-release tablets were unavailable in the Pakistani pharmaceutical market, so the Cardaktin^®^ tablets were selected as reference tablets for dissolution profile comparisons. Rabbits were purchased from animal houses, GCPS, the Faculty of Pharmacy, Gomal University, DIK, and Pakistan. The chemicals used were of analytical grade and were used without further purification.

### 2.2. Tablet Formulation

Formulations of the drug’s API (active pharmaceutical ingredient) were established using different grades of Ethocel polymers (Ethocel 10 premium and Ethocel 10 FP premium) with various D:P (drug:polymer) ratios of 10:3, 10:4, and 10:5, and the D:P was taken as weight by weight (*w*/*w*). Each controlled-release matrix contained 100 mg of the drug (API), which was kept constant in each matrix. Magnesium stearate 0.5% (*w*/*w*) was used as a lubricant, and spray-dried lactose (filler) was used as a filler. Co-excipients such as HPMC, CMC, and starch were added to some selected formulations (D:P of 10:5) by replacing 10% of the fillers. A pilot batch of 110 tablets of each type was formulated. The mixtures of each formulation were made as follows: Each ingredient from each batch was weighed separately using a digital electronic balance (AX-200, Tokyo, Japan). The drug (API) and polymer were mixed using a pestle and mortar. The mixture was added to polybags for further mixing. The filler (spray-dried lactose) was also added, and in the case of matrices with co-excipients such as HPMC, CMC, and starch (10% of the filler), they were also added, and mixing was performed for 15 min. These formulation mixtures were passed through the No. 32 mesh screen, and then the lubricant (magnesium stearate 0.5%) was added, mixed for 10 min, and again passed through the No. 32 mesh screen to ensure uniform mixing. The formulations are shown in Table 1.

### 2.3. Flow Properties of Formulation Mixtures

The flow properties (angle of repose, Carr index, and Hausner ratio) of various physical mixtures of the formulations were determined according to standard methods. The angle of repose specifies the angle established between the height of the powder and its horizontal surface, which measures the amount of friction between the powder particles or granules [28]. To determine the angle of repose, a funnel-and-cone method was employed, in which the funnel was secured with a stand 5 cm above the Petri dish, and each formulation mixture sample was collected in the Petri dish under the funnel to obtain a pile of powder. The diameter of the Petri dish (d) was used to calculate the radius (r) and height (h) of the heap, which were noted using a clean ruler. The radius and height values were fitted to Equation (1). The bulk and tapped volumes of the powder samples were determined to determine the Carr index (%) of the mixtures. The resulting bulk (V_o_) and tapped volumes and the bulk and tapped densities were determined. The bulk and tapped density values matched the Carr index (Equation (2)) [28,29,30]. The Hausner ratio was also determined from Equation (3) by adding the bulk density and tapped density values.

(1) $$\mathrm{Angle}\;\mathrm{of}\;\mathrm{repose}\;(\theta )={tan}^{-1}\frac{h}{r}$$

where h indicates the height of the heap and r represents the radius of the heap [28,29,30].

(2) $$\mathrm{Carr}\;\mathrm{index}\;(\%)=\frac{\rho \;tapped-\rho \;bulk}{\rho \;tapped}$$

(3) $$\mathrm{Hausner}\;\mathrm{ratio}=\frac{\rho \;tapped}{\rho \;tapped}$$

### 2.4. Differential Scanning Calorimetry (DSC) and Fourier-Transform Infrared (FTIR)

DSC was performed according to a standard protocol [31] to check for possible interactions between the drug (API) and excipients in the physical mixtures of the formulations using a DSC device (Mettler Toledo DSC 822E, Greifensee, Switzerland). In the DSC analysis, the drug (API) and 3 mg of the physical mixtures were weighed into an aluminum pan and sealed with a perforated lid. The heat flow rate was 10 °C per minute under a nitrogen gas flow. FTIR analyses were also used to check for possible interactions between the drug (API) and physical mixtures of different formulations using an FTIR device (PerkinElmer, Seer Green, UK). Spectra for the drug (API) and the physical mixtures of the formulations were obtained, and the range was set from 100 to 3500 cm^−1^ for both the drug (API) and its physical mixtures. The samples were individually placed on the instrument table, the handle was gripped to apply some pressure, and the resolution was fixed at 1 cm^−1^.

### 2.5. Tablet Preparation by Direct Compression Method

The matrices (containing 100 mg of losartan potassium) were prepared by the direct compression method. Each type of formulation mixture was directly compressed into matrices using flat surface punches with a diameter of 12 mm on a single-punch tableting machine (Erweka AR 400, Langen, Germany), and the hardness was kept constant (7–10 kg/cm^2^). A total of 110 matrices were prepared in each batch. The matrices from each batch were added to amber glass bottles and stored for future use.

### 2.6. Physical Quality Control Tests of Matrices

The quality of the matrices was assessed by performing different physical and chemical quality control tests according to the standard procedures [28]. The tests were performed in triplicate and expressed as the average and standard deviation (SD) using Microsoft Excel (version 16.78).

#### 2.6.1. Dimensional Tests

The thickness and diameter of 10 matrices in each batch were determined using a Vernier caliper (Erweka, Germany). The mean values of thickness and diameter were determined with the help of Microsoft Excel (version 16.78) and compared with the acceptable ranges of thickness and diameter (2–4 mm and 4–13 mm, respectively) [32].

#### 2.6.2. Hardness Test (Breaking Force)

Ten matrices from each batch were taken randomly, and their hardness was individually determined using a hardness tester (Erweka, Langen, Germany). The average hardness (with standard deviations) was calculated using Microsoft Excel (version 16.78) and compared with the acceptable hardness limit of 5–10 kg/cm^2^ [33].

#### 2.6.3. Friability Test

In the friability test, 20 matrices from each batch were weighed (W1) and placed in a friabilator (Erweka, Langen, Germany) for 4 min at 25 revolutions per min (rpm). The matrices were taken from the friabilator and weighed again (W2) after cleaning, and their percentage friability was determined using Equation (4). The acceptable limit of tablet friability is less than 1.0% [34].

Friability (%) = (W1 − W2/W1) × 100(4)

#### 2.6.4. Weight Variation Test

Twenty matrices were taken randomly from each batch and weighed individually using a digital electronic balance (AX-200, Japan), and averaged weights (with standard deviations) for each type of matrix were calculated using Microsoft Excel (version 16.78). The USP limits for weight variation are tablets with weights above 120 mg and less than 324 mg, where the weight can vary by plus or minus 10% [35].

### 2.7. Chemical Characteristics

The chemical characteristics of the matrices were assessed by assays and in vitro dissolution tests.

#### 2.7.1. Assay

To assay the drugs, 10 matrices were randomly selected from each batch and powdered using a pestle and mortar. A powder equivalent to 100 mg was taken in a volumetric flask (100 mL) and dissolved in phosphate buffer (pH 6.8), and the final volume was made up to 100 mL. The drug was completely dissolved by constant shaking for up to 10 min. The solution was filtered using a membrane filter (0.45 µm) to remove particulate matter from the sample solution. The 10 reference tablets (Cardaktin^®^ tablets containing 100 mg of losartan potassium) were also crushed into powder, and powder equivalent to 100 mg of the drug was taken in a volumetric flask (100 mL) and dissolved in a sufficient volume of phosphate buffer (pH 6.8) by continuous shaking, after which the final volume was made up to 100 mL with the same buffer solution to obtain the standard solution. The absorbances of the sample and standard solution were determined spectrophotometrically at a maximum wavelength of 205 nm for the losartan potassium, and the absorbance values in the assay formula [(sample absorbance/standard absorbance) × 100] were used to complete the drug assay [36].

#### 2.7.2. In Vitro Evaluation

The in vitro evaluation of the matrices of the drug was performed according to USP Method-I [28] (rotating basket method) using the Pharma Test Dissolution Apparatus (D-6312, Hainburg, Germany). The drug was freely soluble (1.0 mg/mL) in phosphate buffer (pH 6.8) and was selected as a dissolution medium. In each of the 8 stations of the dissolution apparatus, phosphate buffer (pH 6.8) was added up to 900 mL. Matrices were placed in the baskets, and the baskets were immersed in the dissolution medium. The rotation speeds of the baskets were maintained at 100 rpm, and the temperature was kept constant at 37 ± 0.5 °C thermostatically. The dissolution studies were carried out for 24 h, and samples of 5 mL were collected at various time intervals (0.5, 1.0, 1.5, 2.0, 3.0, 4.0, 5.0, 6.0, 8.0, 10, 12, 18, and 24 h), after which the same volume of dissolution medium was added to each station as a replacement solution. These samples were filtered through a membrane filter (0.45 µm).

The absorbances were checked spectrophotometrically using a UV–Visible Double-Beam Spectrophotometer (UVIDEC-1610, Shimadzu, Japan) at 205 nm for losartan potassium. The percentage drug release was calculated for each batch from the analytical curve of the drug [37].

### 2.8. Effect of Polymer Particle Size

The influence or effect of particle size was also analyzed because Ethocel is available in granular form (Ethocel 10 premium) and fine particulate form (Ethocel 10 FP premium). The purpose of analyzing the effect of particle size was to determine whether the granular or fine particulate Ethocel was more effective at extending the drug release rates.

### 2.9. Effects of Co-Excipients

The effects of various co-excipients, like HPMC, CMC, and starch, were evaluated to study how these co-excipients modified the drug release rates from the polymeric matrices of the drug. The filler (spray-dried lactose) was replaced (10%) by the co-excipients in some selected formulations (D:P of 10:5) to study their effects on the drug release rate. The co-excipients were used in small amounts in the selected formulations.

### 2.10. Drug Release Kinetics of Matrices

The drug release mechanisms or kinetics were examined by incorporating the in vitro dissolution data of the matrices into different kinetics models, as follows:

1. Zero-order kinetics model [38]:
  W = K_1_t (5)
  where K_1_ is the rate constant, the unit is concentration/time, and t is the time.
2. First-order kinetics model [38]:
  ln (100 − W) = ln100 − K_2_t (6)
  where K_2_ shows the first-order constant and ln100 indicates the initial concentration.
3. Hixson–Crowell erosion model [38]:
  (100 − W)^1/3^ = 100 ^1/3^ − K_3_t (7)
  where (100 − W)^1/3^ is the initial concentration, 100 ^1/3^ is the concentration at time t, and K_3_ is the model constant.
4. Higuchi’s diffusion model [38]:
  W = K_4_t^½^ (8)
  where K_4_ indicates the design variable of the system.
5. Power-law kinetics model [38]:
  M_t_/M_∞_ = K_5_t^n^ (9)
  where M_t_/M_∞_ indicates the fraction of the drug released at time t and K_5_ shows the rate constant, while n is the release exponent; an n-value of 0.5 exhibits quasi-Fickian diffusion, an n-value of >0.5 shows anomalous non-Fickian drug release kinetics, and an n-value of 1 shows a non-Fickian zero-order drug release mechanism [38].

### 2.11. Dissolution Profile Comparison

The percentage drug release profiles of the test matrices were compared with standard reference tablet dissolution profiles by fitting the data into the difference factor (f_1_) to identify where the dissolution profiles of the test tablets showed differences from the reference tablet dissolution profiles. The acceptable limit of the difference factor is 1–15 [36]. Cardaktin^®^ tablets (immediate-release tablets containing 100 mg of losartan potassium) and controlled-release tablets unavailable on the market were taken as reference tablets. Equation (10) is given as follows:

Difference factor (f_1_):

f_1_ = {[∑ t = 1^n^ (R_t-_Rt)]/[∑ t = 1 n Rt] 100 (10)

where n shows the number of pull points.

Wt shows an optional weight factor, with Rt indicating the reference tablet dissolution profile after time t. Tt shows the test tablet dissolution profile at time t [14,39,40].

### 2.12. Effect of Polymer Concentration

The drug amount was kept constant in different formulations, and the polymer amounts were increased according to the drug-to-polymer ratios. The amount of polymer in the matrices was increased from 30 to 50 mg. The effect of the gradually increasing concentration on the drug release rate was investigated to determine whether an increase in polymer concentration retarded the drug release rate or did not affect it. 

### 2.13. In Vivo Evaluation

In the in vivo study, test-optimized matrices (Ethocel 10 FP premium at 10:4) and reference tablets were used, with both types of tablets containing 100 mg of losartan potassium. The study was approved by the Research and Ethical Committee (In-Vivo-Pharm-122, dated 12 March 2019) of the Faculty of Pharmacy, Gomal University, D.I. Khan, and the Protocol of Animal Scientific Procedure Act 1986 was adopted [41]. This study also complied with NIH and FDA guidelines for animal studies [42].

#### 2.13.1. The Animals

Twelve (n = 12) healthy male local-bred albino rabbits (mean age 6.1 months, mean weight 2.7 ± 0.5 kg) were purchased from the animal house of Gomal University. The animals were equally divided into two groups, housed in proper cages (50 × 40 × 30 cm) with 12 h dark and 12 h light, and allowed to drink water ad libitum. This study lasted 4 weeks, so it was completed when the rabbits were 26.94 weeks of age.

#### 2.13.2. Study Design

To determine comparative pharmacokinetic parameters, one test-optimized tablet was given to each rabbit in Group 1, and a reference tablet was given to each rabbit in Group 2.

Before the dose was given, the rabbits were kept fasted for one day, and after the dose, they were again kept fasted with free excess water. The rabbits were shifted to a wooden holder to put the restraints in place, and a dose was given orally with the use of the barrel of a smoothly cut syringe (3 mL) at the needle end to prevent any damage to the rabbit’s oral mucosa or internal injury, allowing for safe and careful administration of the dose. After the safe administration of the tablet, 10 mL of tap water was administered with a 10 mL syringe and an oral tube to mimic human dosing.

#### 2.13.3. Blood Collection, Drug Extraction, and HPLC Analysis

From the marginal ear vein, blood samples of 0.7 mL were collected into heparinized centrifuge tubes before dosing and at different time intervals after dosing (0.5, 1.0, 2.0, 3.0, 4.0, 5.0, 6.0, 8.0, 10, 12, 24, 30, 36, and 48 h), for both the test-optimized matrices and reference tablets during the study period. The collected blood samples were kept to clot for 30 min. The obtained clots were remixed with a sterile wooden stick and kept still in the tubes where the samples were initially collected. Plasma was obtained when the blood samples were centrifuged at 1500 rpm, and the plasma was separated. The plasma sample containing no drug (undosed) was kept blank, and both were placed in the refrigerator at −70 °C (Shel Lab, Sheldon Manufacturing, Inc., Cornelius, OR, USA) until further use. Diethyl ether was incorporated in each of the 1 mL plasma samples, and this mixture was centrifuged at 2500 rpm for 15 to 20 min. The supernatants of about 5 mL were pipetted out, and evaporation occurred at room temperature. Acetonitrile (5 mL) was added to the residue and reconstituted, and the drug concentration was estimated by a well-established rapid HPLC method [43].

HPLC (Agilent 1200, Agilent-Corporation, Waldbronn, Germany) was used with a quaternary pump system and automatic sampler, a PDA (Photodiode Array) detector, and Chem Station Software version E.02.02 (Agilent Corporation, Waldbronn, Germany) for data acquisition. A Phenomenex C-18 column (5 µm; 4.6 mm 250 mm, Luna, Phenomenex, Torrance, CA, USA) was used for chromatographic separation at 25 °C. The mobile phase was prepared and premixed. At a 1:1 ratio, 50 parts of a disodium hydrogen phosphate (30 mM) solution (*v*/*v*) at pH 7 were mixed with 50 parts of acetonitrile (*v*/*v*). The isocratic flow rate was 1.0 mL per min. The rinse solution of acetonitrile and water at 50:50 *v*/*v* was used for rinsing the injector, with an injection volume of 5 µL, and the detection was carried out at a detection wavelength of 220 nm for losartan potassium. The method’s precision, linearity, accuracy, and drug recovery were determined using spiked rabbit serum at 12 to 60 µg/mL concentrations. The intraday and interday variability values were obtained by repeating the injection of quality control (QC) samples. The QC samples were made at 10, 20, and 50 µg/mL, indicating lower, middle, and higher controls.

#### 2.13.4. Determining Pharmacokinetic Parameters

The pharmacokinetic parameters (i.e., the area under the curve (AUC), peak plasma concentration (Cmax), The half-life (t_1/2_), the time required for the drug to reach maximum concentration (T_max_), the area under the curve infinity (AUC_0-inf_), mean residence time (MRT_0–48h_), and clearance (Cl)) were determined using Kinetica version 5.0. Plasma–time curves were also established for the test and formulations. The elimination rate constant (kel) was used to estimate the fractions of the absorbed and unabsorbed drug with the Wagner–Nelson method, as specified in PK-Fit version 2.01 [44]. The percentage of the drug absorbed was calculated using Equation (11):

Percent Absorbed = {(C(_t_)/Ke + AUC (_o-t_)/AUC (_o-∞_)} × 100 (11)

### 2.14. Statistical Analysis

Data were analyzed using GraphPad Prism 9 software (GraphPad Software, Inc., La Jolla, CA, USA). All data are presented as the mean ± standard deviation (SD) and were first checked for normality using the Kolmogorov–Smirnov and D’Agostino–Pearson tests. A one-way ANOVA followed by Tukey’s multiple comparisons test was used to compare the pharmacokinetic parameters of the test-optimized matrices and the reference tablets. The level of significance was set at *p* < 0.05.

The correlation between the optimized tablets’ in vitro and in vivo data was determined by plotting the percentage of drug absorbed (Fa) from the in vivo study against the percentage of in vitro drug release (Fr).

## 3. Results

### 3.1. Flow Characteristics

The flow characteristics of the drug (API) and its formulated physical mixtures were determined (Table 2). Then, the drug (API) powder’s repose angle was 54.34 ± 0.18, its Carr compressibility index was 32.36 ± 0.64, the Hausner ratio was 1.43 ± 0.28, and it showed poor flow characteristics. The angle of repose, Carr compressibility index, and Hausner ratio of the formulated physical mixtures ranged from 28.12 ± 0.36 to 33.98 ± 0.69, from 9.56 ± 0.50 to 14.73 ± 0.38, and from 1.07 ± 0.48 to 1.16 ± 0.18, respectively. The resultant values of all formulation flow parameters were found to range from good to excellent, confirming the values of the good-to-excellent flow of powders (angle of repose: 31–35, Carr compressibility index: 11–15, and Hausner ratio: 1.12–1.18) mentioned in the USP [28].

### 3.2. Results of FTIR and DSC Analyses

In the DSC analysis, the melting point of the drug was found to be 274.5 ± 0.18 °C. The physical mixture with Ethocel-10FP-based selected matrices at a 10:4 ratio showed a melting point of 273.3 ± 0.24 °C (Table 3), indicating a negligible change in the melting points and that they might be compatible. In the FTIR study of the drug (API) and Ethocel-10FP-based matrices in a 10:4 physical mixture, the important functional groups (Table 3) of the drug (API) and the drug in the Ethocel 10FP-based matrices in a 10:4 physical mixture were approximately the same, which might indicate that they were compatible.

### 3.3. Physical Characteristics

The matrices’ thickness, diameter, friability, hardness, and weight were determined (Table 4). The thickness of the matrices ranged from 2.4 ± 0.10 to 2.5 ± 0.43, which is within the USP [28] limits for diameter (2–4 mm). The diameter of the matrices ranged from 8.0 ± 0.09 to 8.0 ± 0.51, which is within the USP [28] limits for diameter (4–13 mm). The friability of the matrices ranged from 0.05 ± 0.07 to 0.25 ± 0.11, which is within the USP [28] limits for friability (<1.0%), while the hardness ranged from 7.8 ± 0.51 to 9.5 ± 0.37, which is within the USP [28] limits for hardness (5–10 kg/cm^2^). The weights of the matrices ranged from 198 ± 0.14 to 203 ± 0.19, which is within the USP [28] limits for weight variation (130 to 324 mg, with 7.5% variation).

### 3.4. Assay

The drug assay was determined for the matrices, which ranged from 98.67 to 99.25% and was within the USP compendial limits for assay (90–110%) [45]. These results are given in Table 5.

### 3.5. Drug Release Profiles and Effects of Polymer (Ethocel) Concentration

**Figure 1:**
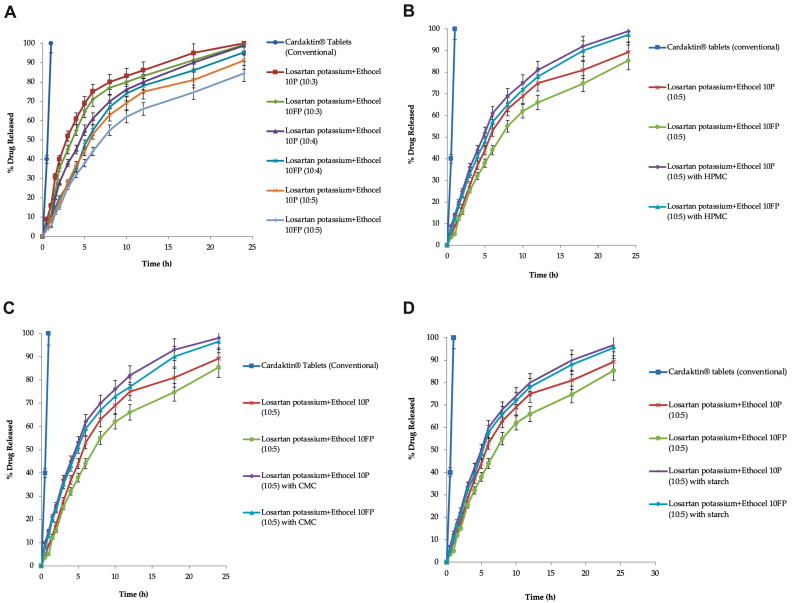
Graphical representation of the drug release profiles of Ethocel grade 10 matrices (**A**) without co-excipients, (**B**) with HPMC as a co-excipient, (**C**) with CMC as a co-excipient, and (**D**) with starch as a co-excipient.

The matrices of the drug were developed by the direct compression method at D:P ratios of 10:3, 10:4, and 10:5, using Ethocel (grade 10) as a rate-altering polymer, and were investigated for drug release for 24 h. The controlled-release matrices with grade 10 Ethocel (Ethocel 10 premium and Ethocel 10FP premium) at D:P ratios of 10:3, 10:4, and 10:5 released 100, 99.34, 98.88, 95.32, 91.14, and 84.39% of the drug in 24 h, respectively (Figure 1A).

### 3.6. Effects of Co-Excipients

In matrices with Ethocel 10 premium and 10FP premium, HPMC enhanced the drug release rate to 98.91 and 97.38%, respectively (Figure 1B). In the case of matrices with Ethocel 10 premium and Ethocel 10FP premium at a D:P ratio of 10:5 with starch as a co-excipient, starch enhanced the drug release rates to 96.66 and 95.49%, respectively, in 24 h (Figure 1D). In matrices with Ethocel 10 premium and 10FP premium with CMC, the drug release rates were enhanced to 96.55 and 98%, respectively (Figure 1C).

### 3.7. Drug Release Kinetics

The drug release data were fitted into different kinetics models. The first-order kinetics model’s r^2^ values, ranging from 0.316 to 0.478, indicated that first-order kinetics might not release the drug. The zero-order kinetics model’s r^2^ values ranged from 0.985 to 0.994, indicating that zero-order kinetics could release the drug. The r^2^ values of the Hixson–Crowell model ranged from 0.891 to 0.986, showing that the drug might be released by swelling or erosion mechanisms. The r^2^ values of Higuchi’s kinetics models ranged from 0.986 to 0.993, indicating that diffusion mechanisms might release the drug. The r^2^ values of power-law kinetics ranged from 0.954 to 0.993, exhibiting linearity in the release patterns, while the n-values ranged from 0.523 to 0.986, showing that the drug might be released by anomalous non-Fickian diffusion (Table 6).

The matrices with Ethocel 10 FP showed more pseudo-zero-order kinetics (n-value: 0.986) (Table 7).

### 3.8. Dissolution Profile Comparisons

The differences in the dissolution patterns were determined using the difference factor (f_1_) [46]. The dissolution patterns of the losartan potassium test matrices based on Ethocel grade 10, with or without co-excipients, when compared with reference standard dissolution profiles, resulted in f_1_ values that ranged from 35.73 to 58.22, indicating that the test matrices’ dissolution patterns were not comparable with those of the reference tablets (Table 8).

### 3.9. Optimized Tablet Formulations

The optimized matrices of losartan potassium were selected based on their drug release patterns and their drug release kinetics. The Ethocel 10FP premium matrices (10:4) were selected as optimized test matrices, as the n (drug release exponent)-value obtained was 0.986 (near 1.0) and was approaching pseudo-zero-order drug release kinetics.

### 3.10. Stability and Reproducibility

A batch of the selected matrices was designed and prepared by direct compression to check their stability and reproducibility. The stability studies were conducted at 40 ± 2 °C, and the relative humidity was 75 ± 5%, in agreement with the ICH guidelines [47]. The physicochemical characteristics (i.e., appearance, thickness, diameter, friability, hardness, weight variation, content uniformity, and dissolution patterns) were determined at zero time (after preparation) and after 3 and 6 months of exposure to accelerated stability conditions. It was noted that the matrices had no significant differences in physicochemical characteristics (Table 9).

### 3.11. In Vivo Studies

A simple, rapid, and reproducible method was adopted to analyze the optimized matrices of the drugs in rabbit serum. At zero time, no peak was obtained, and as the serum spiked, it showed a single peak of losartan potassium. The retention time of the drug was 4.9 min, with 97% sample recovery. The drug calibration curve was obtained in the 12–60 µg/mL range, with an r^2^ value of 0.9998, which showed good linearity. The percentage of drug recovery was determined and ranged from 99.54 ± 0.123 to 101.14 ± 0.024%. The limits of detection and quantification were found to be 0.160 and 0.669 µg/mL, respectively. The precision of the interday and intraday measurements of three samples (25, 45, and 65 µg/mL) ranged from 0.031 to 0.033% and from 0.041 to 0.044%, respectively. All the above parameters complied with the ICH guidelines [47].

#### 3.11.1. Plasma Concentration–Time Curve

**Figure 2:**
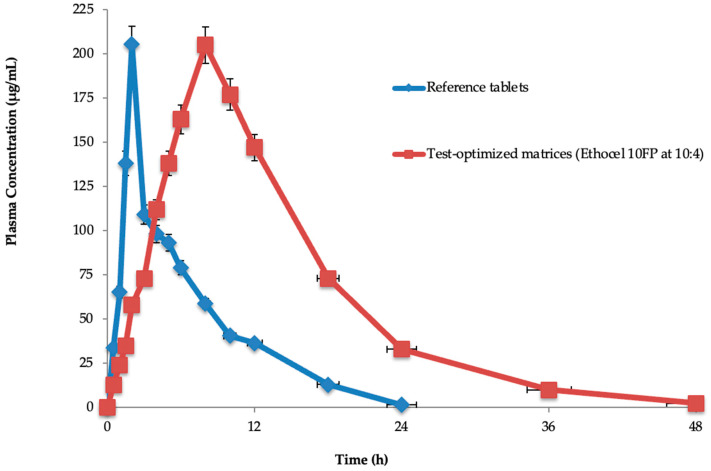
Plasma concentration–time curves of test-optimized matrices and reference tablets.

Losartan potassium plasma concentration (average)–time curves were constructed for the optimized test matrices and the reference tablets. It was noted from the absorption profiles that the test-optimized matrices efficiently extended the drug levels in plasma and were detectable after 36 h of dosing, as compared to the reference standard IR tablets (Figure 2). These results confirm those reported in the literature, where CR matrices could extend the plasma levels of the drug [48,49].

#### 3.11.2. Pharmacokinetic Parameters

The half-life (11.78 ± 0.018 h), T_max_ (2.105 ± 1.131 h), C_max_ (205.98 ± 0.321 μg/mL), AUC_0_ (5931.10 ± 1.232 μg·h/mL), AUC_0-inf_ (7348.46 ± 0.234 μg·h/mL), MRT_0–48h_ (17.34 ± 0.184 h), and Cl (0.002 ± 0.134 mL/min) were found. The reference tablets’ half-life (2.0 ± 0.125 h), T_max_ (8.0 ± 0.091 h), C_max_ (205.98 ± 0.321 μg/mL), AUC_0_ (2534.12 ± 2.29 μg·h/mL), AUC_0-inf_ (6415.32 ± 0.012 μg·h/mL), MRT_0–48h_ (9.98 ± 2.169 h), and Cl (0.005 ± 0.128 mL/min) were also found. In the test-optimized matrices, polymeric materials were present, which significantly (*p* < 0.05) extended the drug’s half-life as compared to the reference tablets, and the other parameters were insignificantly similar (Table 10).

#### 3.11.3. In Vitro and In Vivo Correlation

**Figure 3:**
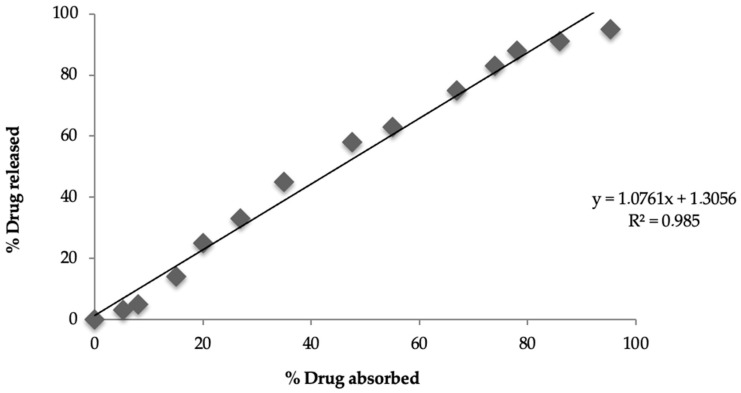
Linearity of in vitro and in vivo correlation between test-optimized matrices.

A correlation was established between the in vitro and in vivo results for the test-optimized matrices, and the resultant r^2^ value was 0.985, indicating a level-A correlation between the percentage of the drug released in vitro and the percentage of the drug absorbed in vivo (Figure 3). A good r^2^ value of 0.9553 was also observed in other in vitro and in vivo studies [50,51,52].

## 4. Discussion

The controlled-release matrices were formulated with the Ethocel grade 10 polymers (Ethocel 10 premium and Ethocel 10FP premium), along with excipients, and they were physically mixed to achieve formulation mixtures. The formulation mixtures’ flow properties (angle of repose, Carr index, and Hausner ratio) were tested using the USP. All of the formulated physical mixtures showed good-to-excellent flow properties (angle of repose 28.12 ± 0.36 to 33.98 ± 0.69, Carr compressibility index 9.56 ± 0.50 to 14.73 ± 0.38, and Hausner ratio 1.07 ± 0.48 to 1.16 ± 0.18), which were found within the USP flow range [28]. The flow properties of the formulated physical mixture of Ethocel 10FP (10:5) with HPMC (angle of repose 28.12 ± 0.36, Carr compressibility index 9.56 ± 0.50, and Hausner ratio 1.07 ± 0.48) were significantly higher (*p* < 0.05) than the flow properties of the remaining formulation mixtures. The formulated physical mixtures showed good-to-excellent flow properties, which might have been due to the proper mixing of the ingredients, and they might have good flow during the compression process due to the addition of the lubricants. Khandai et al. developed carbo-protein polymeric-complex-based sustained-release microspheres of losartan potassium and determined the capacity of the formulation to improve the flowability, compressibility, and tableting properties of losartan potassium; the carbo-protein-based polymeric microspheres helped to sustain the drug release for a prolonged time and increase the flowability, compressibility, and tableting properties of losartan potassium [53]. The current study’s results are also similar to those observed by Vidyadhara et al., who found that the angle-of-repose values for different microcapsules were in the range of 21.6–23.85°, showing the good flow properties of microcapsules. The microcapsule compressibility index was 11.25–15.85%, showing good microcapsule flow properties [54].

The drug (API) and a formulation mixture (Ethocel-10FP-based, at a 10:4 ratio) were subjected to DSC and FTIR analyses to identify any possible drug–excipient interactions, and slight variation was noted in the melting points of the drug (274.5 ± 0.18 °C) and the formulation mixture (273.3 ± 0.24 °C), indicating a negligible change in the melting points and that they might be compatible. These results of the DSC study confirmed the findings of Vidyadhara et al., who reported a lack of drug–polymer interaction in the DSC studies of the drug (API) and formulation mixtures [54]. The FTIR analysis showed that the same functional groups (OH, C–H aliphatic stretching, C=N stretching, and C=C stretching) were found for the drug (API) and the formulation mixture (Ethocel-10FP-based, at a 10:4 ratio), with slight variations in wave numbers, which might indicate that they were compatible. These results (FTIR) confirm other authors’ findings that no possible interaction was found between the drug and excipients in FTIR studies, while compatibility was observed due to the main functional groups (OH, C–H, and C=N) being intact for the pure drug and the formulation powders [55].

The matrices were subjected to physical quality control tests (thickness, diameter, friability, hardness, and weight determined for the matrices). The thickness of the matrices ranged from 2.4 ± 0.10 to 2.5 ± 0.43, the diameter of the matrices ranged from 8.0 ± 0.09 to 8.0 ± 0.51, the friability ranged from 0.05 ± 0.07 to 0.25 ± 0.11, the hardness ranged from 7.8 ± 0.51 to 9.5 ± 0.37, and the weight of the matrices ranged from 198 ± 0.14 to 203 ± 0.19, all of which were within the USP limits (thickness: 2–4 mm, diameter: 4–13 mm, hardness: 5–10 kg/cm^2^, friability: <0.1%, weight: 130 to 324 mg with 7.5% variation) for the tablets’ physical tests, and this might have been because the compression process was handled carefully. These results were similar to those of the authors [25], who also found the physical characteristics of their tablets to be within an acceptable range due to proper handling during the tableting process.

The drug assay was performed for the matrices, the results of which ranged from 98.67 to 99.25% and were within the USP compendial limits (90–110%) [55]. This might have been due to the proper mixing and blending of the formulation ingredients, and the drug contents might have been uniformly distributed in the matrices. Masood et al. also reported that the drug in a controlled-release hydrogel was within the compendial limits (90–110%), which could have been due to the uniform distribution of the drug in the hydrogels [56]. The matrices were investigated for drug release for 24 h; it was found that the drug release was extended by 24 h. The controlled-release matrices with grade 10 Ethocel (Ethocel 10 premium and Ethocel 10FP premium) at D:P ratios of 10:3, 10:4, and 10:5 released 100, 99.34, 98.88, 95.32, 91.14, and 84.39% of the drug in 24 h, respectively. It was noted that as all of the matrices increased the polymer concentration at different D:P ratios, the drug release rates were also extended, and this might have been due to the increased polymer concentration providing more hindrance to penetration of the dissolution medium and, as a result, extending the drug release rates. Previously, Dash and Verma developed a losartan potassium polymeric matrix sustained-release tablet to extend the drug release up to a time of 24 h at a determined rate, and the in vitro dissolution profile for batch B4 was designed with a blend of HPMC K4M (67.2 mg), HPMC K200M (90 mg), and Eudragit RSPO (112.5 mg), where the drug release was about 94–98% [57]. Wani et al. evaluated polymers HPMC K4M/ethyl cellulose-based expandable film (an oral sustained-release dosage form) loaded with losartan potassium, appropriate for extended gastric retention time and extended drug release over 12 h, which could be due to the HPMC K4M polymer’s swelling, diffusion, and erosion, along with the ethyl cellulose’s reduced water penetration, which might have caused drug release retardation [58]. The authors Chithaluru et al. used the USP XXII dissolution apparatus for in vitro drug release experiments, indicating that the formulation (losartan potassium with sodium CMC and ethyl cellulose) produced a sustained effect, with 74.19% release, and due to diffusion along with erosion of the drug from the polymers’ swelling [24]. These results confirmed the findings of other authors, such as Jan et al., who also observed retardation of drug release rates with increasing polymer concentrations when studying the effect of polymer concentration on the drug release rates of ibuprofen [59]. The effects of the co-excipients (HPMC, CMC, and starch) on the drug release rates were checked, and they enhanced the drug release rates. In matrices with Ethocel 10 premium and 10FP premium, HPMC enhanced the drug release rates to 98.91 and 97.38%, respectively. The matrices with Ethocel 10 premium and Ethocel 10FP premium at a D:P ratio of 10:5 with starch as a co-excipient enhanced the drug release rates to 96.66 and 95.49%, respectively, in 24 h. In matrices with Ethocel 10 premium and 10FP premium with CMC, the drug release rates were enhanced to 96.55 and 98%, respectively. This might have been due to swelling of the co-excipients (HPMC, CMC, and starch), which might have caused an increase in internal osmotic pressure in the matrices, enhancing the drug release rates. Another possible reason might be the disintegrating behaviors of the co-excipients when used in small amounts (i.e., 10% of filler). Khan and Meidan (2007) [60] checked the effects of co-excipients on the drug release and found that the co-excipients enhanced the drug release rate, possibly due to swelling of CMC when exposed to water, along with the internal osmotic pressure, which was enhanced by the burst release of the drug. Their findings were similar to the results of the current study. Jan et al. (2012) observed a similar burst release of the drug for ibuprofen controlled-release matrices when co-excipients were added to enhance the drug release rates [59]. The influence of numerous co-excipients on ibuprofen–Ethocel controlled-release matrices with a D: P ratio of 10:3 was checked to increase the drug release rates. They found that the co-excipients increased the drug release rates, which could have been due to swelling, rupture of the polymer, and increased osmotic pressure in the matrices, similar to the current results. Drug release rate enhancement by co-excipients (HMPC, CMC, and starch) was also observed by Akhlaq et al. (2011) [61], who added co-excipients in small amounts to ibuprofen–Eudragit-S-100-based matrices and found disintegration, swelling, and increasing osmotic pressure inside the matrices containing the co-excipients, which conformed to the present results. The drug was released by the matrices through Fickian diffusion, erosion/swelling (Hixson–Crowell model r^2^ values: 0.891 to 0.986), and anomalous non-Fickian diffusion (power-law model n-values: 0.523 to 0.986), but not by first-order kinetics (first-order r^2^ values: 0.216 to 0.478).

The matrices with Ethocel 10 FP showed more pseudo-zero-order kinetics (power-law model n-value: 0.986) than the remaining matrices (0.523 to 0.858). This might have been due to the hydrophobic nature of the Ethocel grade 10 polymers, which might reduce the penetration of the dissolution medium and retard the drug release rates in a controlled manner rather than burst drug release. The other release mechanisms might have been due to swelling of the polymeric matrices, diffusion of drugs from the swelling matrices, and erosion of the matrix. These current results are similar to those of Wahab et al. [62], who observed that ketoprofen polymeric controlled-release tablets released the drug with anomalous non-Fickian diffusion, swelling, and erosion mechanisms, and their power-law model’s n-values were 0.596 and 0.784, indicating a concentration-independent release of the drug. Ain et al. applied kinetics models to the in vitro release data of the formulation, and it was found that formulation F13 exhibited zero-order release kinetics (n-values: 0.9242–0.9421) due to matrix erosion and swelling [63]. Thus, their drug release from the prepared tablets was controlled by more than one mechanism, i.e., polymer swelling followed by drug diffusion from the swelled polymer and the tablets’ gradual erosion, and these findings conformed to the current results.

The test matrices based on Ethocel grade 10 (D:P 10:5) with no co-excipient or without the co-excipients’ dissolution patterns, when compared with the reference standard dissolution profiles, resulted in f_1_ values that ranged from 35.73 to 58.22, indicating that the test matrices’ dissolution patterns were not comparable with the reference tablets’ dissolution profiles because these results were not found to have acceptable ranges or limits for f_1_ (1–15). This might have been because the test matrices contained polymers that extended the drug release rates (24 h), but the reference tablets were immediate-release tablets, and burst release of the drug occurred, which might have led to differences in their dissolution patterns. Hossain et al. determined the difference factor (f_1_) of all the formulations (F-1 to F-5) to compare their dissolution profiles with that of the innovator brand, and the f_1_ values ranged from 1.76 to 40.22. Formulation F-3′s f_1_ value was 1.76, which showed that the dissolution profiles of F-3 and the innovator brand were different, and their results contradict the current finding of f_1_ (35.73 to 58.22) [64]. Ghosh and Barik compared the dissolution profile of the aceclofenac sustained-release formulation with that of the Aroff 200 mg SR tablets by applying f_1_, and the f_1_ value was 2.44, which is within the acceptable range of f_1_, contradicting the current study’s results for f1 (35.73 to 58.22) [65].

The physicochemical characteristics of the selected matrices were found to be stable when exposed to accelerated stability conditions (at 40 ± 2 °C and relative humidity of 75 ± 5%) for 6 months, and no significant (*p* < 0.05) differences were noted in their physicochemical characteristics. The optimized formulation was exposed to stability conditions as per the ICH guidelines at 25 ± 2 °C and 60 ± 5% relative humidity, as well as accelerated stability conditions (40 ± 2 °C and 75 ± 5% relative humidity). Viveksarathi et al. found that the optimized tablets did not show any significant variation (*p* < 0.05) in average weight, hardness, drug release, or drug content in any of the tablets before and after the stability studies, similar to the results of the current stability study [66].

The half-life (11.78 ± 0.018 h), T_max_ (2.105 ± 1.131 h), C_max_ (205.98 ± 0.321 μg/mL), AUC_0_ (5931.10 ± 1.232 μg·h/mL), AUC_0-inf_ (7348.46 ± 0.234 μg·h/mL), MRT_0–48h_ (17.34 ± 0.184 h), and Cl (0.002 ± 0.134 mL/min) were found for the test-optimized matrices. The reference tablets’ half-life (2.0 ± 0.125 h), T_max_ (8.0 ± 0.091 h), C_max_ (205.98 ± 0.321 μg/mL), AUC_0_ (2534.12 ± 2.29 μg·h/mL), AUC_0-inf_ (6415.32 ± 0.012 μg·h/mL), MRT_0–48h_ (9.98 ± 2.169 h), and Cl (0.005 ± 0.128 mL/min) were also found. In the test-optimized matrices, polymeric materials were present, which significantly (*p* < 0.05) extended the drug’s half-life (11.78 ± 0.018 h) compared to the reference tablets (2.0 ± 0.125 h), while the other parameters were insignificantly similar. This might have been because the polymers extended the in vivo drug release from the optimized matrices. Rahamathulla et al. evaluated pure losartan potassium and an optimized formulation (F3), and the C_max_ after oral administration was 298.4 ± 12.45 ng mL^−1^ for pure losartan potassium and 148.4 ± 15.86 ng mL^−1^ for F3 [67]. The T_max_ for pure losartan potassium was 1.5 h, and for F3 it was 4.1 h. The obtained AUC_0–24_ values were 928.12 ± 51.67 and 1382.40 ± 112.23 ng mL^−1^ for the pure drug and F3, respectively. The MRT was increased from 4.223 ± 0.07 h for an oral solution to 18.92 ± 0.21 h with the sustained-release floating matrix tablet formulations. Their statistical analysis by *t*-test indicated that the pure losartan potassium and the optimized formulations differed significantly (*p* < 0.05), and their results contradict the current study’s findings of pharmacokinetic parameters, except for half-life. Chen et al. prepared and evaluated a losartan potassium and verapamil hydrochloride compound transdermal drug delivery system (TDDS) [68]. Pharmacokinetic analysis indicated that the AUC_0–t,_ and MRT values determined for the patches were significantly better than those achieved after oral administration. Moreover, the plasma concentrations of losartan potassium and verapamil hydrochloride remained above 2 μg/mL for 24 h, which is an effective therapeutic amount. The AUC_0–t_ ratios relating to the TDDS of losartan potassium–verapamil hydrochloride and oral administration were evaluated, and it was noted that the TDDS’s bioavailability reached 338.51%, which was higher than that of the orally administered dosage form, and their results contradict the current study’s results on pharmacokinetic parameters (AUC_0–t_, MRT, and AUC_0–t_). Selvadurai et al. statistically compared the pharmacokinetic parameters of two different drug formulations of losartan potassium, which resulted in significantly different results, contradicting the current findings on C_max_, AUC_0–t_, and AUC_o–∞_ [50].

A correlation was established between in vitro drug release (24 h) and in vivo drug absorption (24 h) for the test-optimized matrices, and the resultant r^2^ value was 0.985, indicating a level-A correlation between the percentage drug release in vitro (95.32%) and the percentage drug absorption (95%) in vivo. The current in vivo study was conducted in albino rabbits; however, it cannot be ruled out that human physiology might affect drug absorption and IVIVC. Qin et al. attempted to find the relationship between plasma concentration and drug release from selected pregabalin tablets, and they found a good in vivo–in vitro correlation (R^2^ = 0.97 in fed conditions), showing that the in vivo response can be predicted by in vitro drug release, and their results for IVIVC are consistent with those of the current study [69].

## 5. Conclusions

The present study prepared controlled-release matrices of losartan potassium with the polymer Ethocel grade 10 (Ethocel 10 premium and Ethocel 10FP premium). It was observed in the FTIR study that there was no or negligible incompatibility between the drug and the excipients. The flow properties were found to be in the good-to-excellent range for USP-2007, and the tablets’ physical characteristics were also in the acceptable range for USP-2007. All of the matrices extended the drug release to 24 h, and the drug was released by anomalous non-Fickian diffusion. Other drug release mechanisms, i.e., erosion and swelling, were noted, followed by the Hixson–Crowell kinetics and Higuchi’s models. The Ethocel 10 FP formulation at a D:P ratio of 10:4 exhibited pseudo-zero-order kinetics (n-value of 0.986) when subjected to the power-law kinetics model. The dissolution profiles of the reference tablets and test matrices were compared using f_1_, and the f_1_ values were not within acceptable limits (1–15). The half-life (11.78 ± 0.018 h), T_max_ (2.105 ± 1.131 h), C_max_ (205.98 ± 0.321 μg/mL), AUC_0_ (5931.10 ± 1.232 μg·h/mL), AUC_0-inf_ (7348.46 ± 0.234 μg·h/mL), MRT_0–48h_ (17.34 ± 0.184 h), and Cl (0.002 ± 0.134 mL/min) were found. The reference tablets’ half-life (2.0 ± 0.125 h), T_max_ (8.0 ± 0.091 h), C_max_ (205.98 ± 0.321 μg/mL), AUC_0_ (2534.12 ± 2.29 μg·h/mL), AUC_0-inf_ (6415.32 ± 0.012 μg·h/mL), MRT_0–48h_ (9.98 ± 2.169 h), and Cl (0.005 ± 0.128 mL/min) were also found. In the test-optimized matrices, polymeric materials significantly extended the drug’s half-life compared to the reference tablets, while the other parameters were unaffected. Level-A in vitro–in vivo correlation was established, with an r^2^ of 0.985. It can be concluded from this study that controlled-release matrices could improve patient compliance with once-a-day dosing and improve therapeutic outcomes.

## Figures and Tables

**Table 1:** Composition of losartan potassium controlled-release matrices.

Drug-to-Polymer Ratio	Drug (mg)	Spray-Dried Lactose (mg)	Magnesium Stearate (0.5%, mg)	Ethocel 10 FP Premium (mg)	Ethocel 10 Premium (mg)	Co-Excipient HPMC (10% of Filler)	Co-Excipient CMC (10% of Filler)	Co-Excipient Starch (10% of Filler)
10:3	100	69	1.0	---	30	---	---	---
10:3	100	69	1.0	30	---	---	---	---
10:4	100	59	1.0	---	40	---	---	---
10:4	100	59	1.0	40	---	---	---	---
10:5	100	49	1.0	---	50	---	---	---
10:3	100	49	1.0	50	---	---	---	---
10:5	100	44.1	1.0	---	50	4.9	---	---
10:5	100	44.1	1.0	50	---	4.9	---	---
10:5	100	44.1	1.0	---	50	---	4.9	---
10:5	100	44.1	1.0	50	---	---	4.9	---
10:5	100	44.1	1.0	---	50	---	---	4.9
10:5	100	44.1	1.0	50	---	---	---	4.9

**Table 2:** Flow characteristics of the formulations based on Ethocel grade 10.

Formulations	Angle of Repose (°)	Carr Index (%)	Hausner Ratio
Ethocel 10P (10:3)	33.42 ± 0.55	14.32 ± 0.23	1.16 ± 0.18
Ethocel 10FP (10:3)	32.20 ± 0.65	13.56 ± 0.48	1.15 ± 0.53
Ethocel 10P (10:4)	30.13 ± 0.78	11.65 ± 0.52	1.12 ± 0.27
Ethocel 10FP (10:4)	33.98 ± 0.69	14.73 ± 0.38	1.15 ± 0.56
Ethocel 10P (10:5)	31.25 ± 0.46	12.78 ± 2.34	1.14 ± 0.49
Ethocel 10FP (10:5)	32.77 ± 0.63	13.58 ± 1.12	1.15 ± 1.16
Ethocel 10P (10:5) + HPMC	30.51 ± 0.28	11.68 ± 0.33	1.14 ± 0.18
Ethocel 10FP (10:5) + HPMC	28.12 ± 0.36	9.56 ± 0.50	1.07 ± 0.48
Ethocel 10P (10:5) + CMC	29.71 ± 0.13	10.84 ± 0.15	1.10 ± 0.23
Ethocel 10FP (10:5) + CMC	30.14 ± 0.32	11.14 ± 0.82	1.14 ± 0.30
Ethocel 10P (10:5) + starch	30.26 ± 1.73	11.25 ± 0.40	1.13 ± 0.45
Ethocel 10FP (10:5) + starch	30.58 ± 0.24	11.76 ± 0.14	1.14 ± 0.28

**Table 3:** FTIR and DSC analysis interpretation.

**FTIR Analysis**		
**Formulations**	**Wave Number**	**Functional Groups**
Drug (API)	3160.47 cm^−1^	O–H
	2906.97 cm^−1^	C–H aliphatic stretching
	1630.25 cm^−1^	C=N stretching
	1573.75 cm^−1^	C=C stretching
Ethocel-10FP-based matrices in a 10:4 formulation mixture	3160.86 cm^−1^	O–H
	2906.99 cm^−1^	C–H aliphatic stretching
	1631.08 cm^−1^	C=N stretching
	1573.96 cm^−1^	C=C stretching
**DSC Analysis**		
**Formulations**		**Melting Points**
Drug (API)		274.5 ± 0.18 °C
Ethocel-10FP-based matrices in a 10:4 formulation mixture		274.27 ± 0.24 °C

**Table 4:** Physical characteristics of matrices.

Formulations	Thickness (mm, n = 10)	Diameter (mm, n = 10)	Friability (%, n = 20)	Hardness (kg/cm^2^, n = 10)	Weight Variation (mg, n = 20)
Ethocel 10P (10:3)	2.5 ± 0.17	8.0 ± 0.28	0.21 ± 0.12	7.8 ± 0.51	202 ± 0.26
Ethocel 10FP (10:3)	2.4 ± 0.23	8.0 ± 0.37	0.19 ± 0.16	9.5 ± 0.37	201 ± 0.25
Ethocel 10P (10:4)	2.5 ± 0.33	8.0 ± 0.19	0.23 ± 0.23	8.5 ± 0.46	203 ± 0.19
Ethocel 10FP (10:4)	2.4 ± 0.26	8.0 ± 0.17	0.18 ± 0.22	9.2 ± 0.58	202 ± 0.14
Ethocel 10P (10:5)	2.5 ± 0.18	8.0 ± 0.34	0.24 ± 0.19	8.3 ± 0.46	200 ± 0.26
Ethocel 10FP (10:5)	2.5 ± 0.26	8.0 ± 0.16	0.17 ± 0.35	9.5 ± 0.35	202 ± 0.17
Ethocel 10P (10:5) with HPMC	2.5 ± 0.43	8.0 ± 0.09	0.09 ± 0.25	8.8 ± 0.07	198 ± 0.14
Ethocel 10FP (10:5) with HPMC	2.4 ± 0.10	8.0 ± 0.20	0.13 ± 0.34	9.8 ± 0.09	200 ± 0.33
Ethocel 10P (10:5) with CMC	2.5 ± 0.31	8.0 ± 0.51	0.05 ± 0.07	8.5 ± 0.28	203 ± 0.11
Ethocel 10FP (10:5) with CMC	2.4 ± 0.28	8.0 ± 0.14	0.25 ± 0.11	9.3 ± 0.33	202 ± 0.44
Ethocel 10P (10:5) with starch	2.5 ± 0.41	8.0 ± 0.19	0.13 ± 0.21	8.5 ± 0.21	200 ± 0.18
Ethocel 10FP (10:5) with starch	2.4 ± 0.13	8.0 ± 0.30	0.15 ± 0.25	9.3 ± 0.32	199 ± 0.14

**Table 5:** Assay of the matrices.

Formulations	Drug Assay (%, mean of n = 10)
Ethocel 10P (10:3)	99.11
Ethocel 10FP (10:3)	99.06
Ethocel 10P (10:4)	98.67
Ethocel 10FP (10:4)	99.14
Ethocel 10P (10:5)	98.77
Ethocel 10FP (10:5)	98.70
Ethocel 10P (10:5) with HPMC	99.06
Ethocel 10FP (10:5) with HPMC	99.25
Ethocel 10P (10:5) with CMC	98.58
Ethocel 10FP (10:5) with CMC	98.85
Ethocel 10P (10:5) with starch	98.57
Ethocel 10FP (10:5) with starch	98.83

**Table 6:** Drug release kinetics of the matrices based on Ethocel grade 10.

First-Order Kinetics		Zero-Order Kinetics		Hixson–Crowell Erosion Model		Higuchi’s Diffusion Model		Power-Law Model		
k_1_ ± SD	r^2^	k_2_ ± SD	r^2^	k_3_ ± SD	r^2^	k_4_ ± SD	r^2^	k_5_ ± SD	r^2^	n
Ethocel 10 premium (10:3) matrices										
(−)0.304 ± 0.118	0.353	8.754 ± 0.878	0.966	0.262 ± 0.117	0.891	7.569 ± 0.163	0.965	0.021 ± 0.056	0.965	0.605
Ethocel 10FP premium (10:3) matrices										
(−)0.134 ± 0.124	0.391	8.677 ± 0.435	0.977	0.238 ± 0.231	0.926	7.556 ± 0.268	0.977	0.073 ± 0.176	0.976	0.721
Ethocel 10 premium (10:4) matrices										
(−)0.193 ± 0.172	0.382	8.453 ± 0.651	0.987	0.212 ± 0.007	0.929	7.331 ± 0.054	0.988	0.037 ± 0.085	0.954	0.986
Ethocel 10FP premium (10:4) matrices										
(−)0.126 ± 0.078	0.459	6.433 ± 0.675	0.990	0.154 ± 0.123	0.903	6.544 ± 0.875	0.990	0.227 ± 0.442	0.993	0.632
Ethocel 10 premium (10:5) matrices										
(−)0.128 ± 0.334	0.463	6.345 ± 0.177	0.993	0.161 ± 0.077	0.957	6.372 ± 0.238	0.993	0.237 ± 0.319	0.968	0.795
Ethocel 10FP premium (10:5) matrices										
(−)0.089 ± 0.134	0.429	5.568 ± 1.321	0.985	0.132 ± 0.365	0.907	5.842 ± 1.323	0.987	1.188 ± 3.237	0.967	0.858

**Table 7:** Drug release kinetics of losartan potassium matrices based on Ethocel grade 10 with co-excipients (HPMC, CMC, and starch) at D:P ratios of 10:5.

First-Order Kinetics		Zero-Order Kinetics		Hixson–Crowell Erosion Model		Higuchi’s Diffusion Model		Power-Law Model		
k_1_ ± SD	r^2^	k_2_ ± SD	r^2^	k_3_ ± SD	r^2^	k_4_ ± SD	r^2^	k_5_ ± SD	r^2^	N
Ethocel 10 premium (10:5) matrices with HPMC										
(−)0.416 ± 0.323	0.356	5.125 ± 0.326	0.990	0.323 ± 0.345	0.927	5.369 ± 0.325	0.993	0.015 ± 0.027	0.977	0.537
Ethocel 10FP premium (10:5) matrices with HPMC										
(−)0.332 ± 0.335	0.347	5.341 ± 0.387	0.992	0.329 ± 0.454	0.948	6.463 ± 0.565	0.993	0.010 ± 0.006	0.939	0.585
Ethocel 10 Premium (10:5) matrices with CMC										
(−)0.352 ± 0.223	0.346	5.345 ± 0.144	0.987	0.351 ± 0.528	0.865	5.321 ± 0.252	0.993	0.007 ± 0.011	0.962	0.523
Ethocel 10FP premium (10:5) matrices with CMC										
(−)0.335 ± 0.273	0.316	5.345 ± 0.285	0.994	0.338 ± 0.2548	0.919	5.743 ± 0.732	0.995	0.013 ± 0.025	0.957	0.540
Ethocel 10 premium (10:5) matrices with Starch										
(−)0.346 ± 0.198	0.454	6.120 ± 0.634	0.984	0.328 ± 0.426	0.967	6.439 ± 0.274	0.986	0.012 ± 0.029	0.948	0.548
Ethocel 10FP premium (10:5) matrices with Starch										
(−)0.338 ± 0.123	0.478	6.356 ± 0.345	0.986	0.332 ± 0.244	0.969	6.564 ± 0.128	0.989	0.032 ± 0.037	0.915	0.619

**Table 8:** Dissolution pattern comparisons between the matrices and reference tablets (mean n = 3).

Test Matrices Versus Reference Tablets	f_1_ Values
Ethocel 10P (10:3) matrices versus reference tablets	35.73
Ethocel 10FP (10:3) matrices versus reference tablets	40.03
Ethocel 10P (10:4) matrices versus reference tablets	40.72
Ethocel 10FP (10:4) matrices versus reference tablets	50.13
Ethocel 10P (10:5) matrices versus reference tablets	52.96
Ethocel 10P (10:5) matrices versus reference tablets	58.22
Ethocel 10P (10:5) with HPMC matrices versus reference tablets	45.41
Ethocel 10FP (10:5) with HPMC matrices versus reference tablets	48.27
Ethocel 10P (10:5) with CMC matrices versus reference tablet	44.51
Ethocel 10FP (10:5) with CMC matrices versus reference tablets	47.23
Ethocel 10P (10:5) with starch matrices versus reference tablets	47.12
Ethocel 10FP (10:5) with starch matrices versus reference tablets	49.31

**Table 9:** Stability studies of the selected matrices under accelerated temperature conditions.

Test Period (Months)	Appearance	Diameter (n = 10, Mean ± SD)	Thickness (n = 10, Mean ± SD)	Friability (n = 20, Mean ± SD)	Hardness (n = 10, Mean ± SD)	Weight Variation (n = 10, Mean ± SD)	Content Uniformity (n = 10, Mean ± SD)	% Drug Release at 24 h (n = 10, Mean ± SD)
0	Whitish	2.4 ± 0.11	8.0 ± 0.15	0.16 ± 0.13	9.3 ± 0.26	201 ± 0.05	98.67 ± 0.14	91.98 ± 0.13
3	Whitish	2.4 ± 0.35	8.0 ± 0.23	0.15 ± 0.35	9.2 ± 0.17	201 ± 0.12	98.58 ± 0.08	90.99 ± 0.16
6	Whitish	2.4 ± 0.54	8.0 ± 0.37	0.14 ± 0.35	9.1 ± 0.17	201 ± 0.58	98.43 ± 0.02	90.45 ± 0.21

Note: one-way analysis of variance (ANOVA) was applied at a *p* < 0.05 significance level.

**Table 10:** Pharmacokinetic parameters of optimized and reference tablets in rabbit sera (n = 12, mean ± SD).

Pharmacokinetic Parameters	Test-Optimized Matrices	Reference Tablets
Half-life t_1/2_ (h)	11.78 ± 0.018	2.0 ± 0.125
Time of maximum plasma concentration T_max_ (h)	8.0 ± 0.091	2.105 ± 1.131
Maximum plasma concentration C_max_ (μg/mL)	204.45 ± 0.289	205.98 ± 0.321
Area under the curve AUC_0_ (μg·h/mL)	5931.10 ± 1.232	2534.12 ± 2.29
Area under the curve AUC_0-inf_ (μg·h/mL)	7348.46 ± 0.234	6415.32 ± 0.012
Mean residence time: MRT_0–48h_ (h)	17.34 ± 0.184	9.98 ± 2.169
Clearance (Cl) (mL/min)	0.002 ± 0.134	0.005 ± 0.128

Parameter values indicated significant differences (*p* < 0.05) between the means of the test-optimized matrices of losartan potassium and the reference tablets.

## Data Availability

The data presented in this study are available in this article.
